# Small fragments of hyaluronan are increased in individuals with obesity and contribute to low-grade inflammation through TLR-mediated activation of innate immune cells

**DOI:** 10.1038/s41366-022-01187-z

**Published:** 2022-07-27

**Authors:** Mònica Romo, Cristina López-Vicario, Noelia Pérez-Romero, Mireia Casulleras, Ana Isabel Martínez-Puchol, Belén Sánchez, Roger Flores-Costa, José Alcaraz-Quiles, Marta Duran-Güell, Ainitze Ibarzábal, Juan José Espert, Joan Clària, Esther Titos

**Affiliations:** 1 Biochemistry and Molecular Genetics Service, Hospital Clínic-IDIBAPS, Barcelona, Spain; 2 CIBERehd, Madrid, Spain; 3 Gastroenterology Department, Hospital Universitari Mútua de Terrassa, Terrassa, Spain; 4 Molecular Biology CORE, Biomedical Diagnostic Center, Hospital Clínic, Barcelona, Spain; 5 Gastrointestinal Surgery Department, Hospital Clínic-IDIBAPS, Barcelona, Spain; 6 EF-Clif and Grifols Chair, Barcelona, Spain; 7 Department of Biomedical Sciences, University of Barcelona, Barcelona, Spain

**Keywords:** Obesity, Obesity

## Abstract

**Background and aim:**

Extracellular matrix (ECM) components released during excessive fat mass expansion are considered potential endogenous danger/alarm signals contributing to innate immune system activation. The aim of the current study was to specifically measure plasma levels of low molecular weight (LMW) hyaluronan (HA) and to evaluate its role as pro-inflammatory damage-associated molecular pattern (DAMP) on leukocyte response in the context of human obesity.

**Subjects and methods:**

Participants were selected according to their body mass index (BMI, kg/m^2^) as non-obese (BMI < 29.9, *n* = 18) and obese (BMI > 29.9, *n* = 33). Plasma samples were size-dependent fractionated using ion-exchange chromatography to specifically obtain LMW HA fractions that were subsequently quantified by ELISA. Cell incubation experiments with synthetic HA molecules were performed on freshly Ficoll-isolated neutrophils (PMN) and peripheral blood monocytes (PBMC). Leukocyte and adipose tissue gene expression was assessed by real-time PCR and NF-κB activation by western blot. Plasma cytokine levels were measured by fluorescent bead-based (Luminex) immunoassay.

**Results:**

We observed a statistically significant increase in the circulating levels of HA fragments of LMW in individuals with obesity which were consistent with significant up-regulated expression of the LMW HA synthesizing enzyme hyaluronan synthase-1 (HAS-1) in obese adipose tissue. Gene expression assessment of HA receptors revealed up-regulated levels for TLR2 in both obese PMN and PBMC. Synthetic HA molecules of different sizes were tested on leukocytes from healthy donors. LMW HA fragments (15–40 kDa) and not those from intermediate molecular sizes (75–350 kDa) induced a significant up-regulation of the expression of major pro-inflammatory cytokines such as IL-1β, MCP-1 and IL-8 in PBMC. Importantly, LMW HA was able to induce the phosphorylation of IKK α/β complex supporting its pro-inflammatory role through NF-κB activation.

**Conclusion:**

Circulating LMW HA molecules are elevated in obesity and may play an important role in triggering low-grade inflammation and the development of metabolic complications.

## Introduction

During the past decades, studies on tissue dynamics of hyaluronan (HA), the most abundant non-sulfated glycosaminoglycan (GAG) in the extracellular matrix (ECM), have revealed important clues to understand its role in health and disease. Firstly, HA is known to be elevated in several pathological conditions where the structure and organization of the ECM is compromised such as joint disease [1], liver fibrosis [2], diabetes [3], myocardial damage [4] and certain types of cancers [5]. Measuring its circulatory levels is used as a biomarker to monitor tissue damage [6] and for instance, plasmatic total content of HA is used within different algorithms to predict liver fibrosis [7]. Secondly, distinct functional properties have been ascribed according to its molecular size [8]. Specifically, the presence of HA as a high-molecular-weight (HMW) polymer (>10^3^ kDa) prevents tissue from dehydration and structural compressive damage and has been shown to be anti-inflammatory, anti-angiogenic, and immunosuppressive [9]. In contrast, lower molecular weight (LMW) species (<500 kDa) have been shown to induce the expression of pro-inflammatory cytokines, chemokines, and growth factors and participate in cancer progression [10, 11]. By the direct interaction with specific cell membrane receptors such as its canonic glycoprotein cell adhesion molecule CD44 or the lymphatic vessel endothelial HA receptor-1 (LYVE-1) and indirect interactions with Toll-like receptors (TLR) 2 and 4, HA molecules activate cell signaling cascades and modulate cell responses to tissue injury [12–15]. The homeostatic HA turnover is controlled by three distinct multipass transmembrane HA synthases (HAS1, 2 and 3) and three HA degrading enzymes or hyaluronidases (HYAL1, 2 and 3). Tissue pathological conditions may alter HA metabolism giving rise to increased production of small HA fragments by both enzymatic [16] and non-enzymatic circuits dependent on reactive oxygen species (ROS) which are able to break down HA molecules into lower molecular weight GAG chains [17].

In obesity, dramatic expansion of adipose tissue during weight gain disrupts tissue functional and structural homeostasis by initially altering local oxygen supply and consequently inducing cellular stress processes, inflammation, and severe deregulation of ECM component. This situation entails an important efflux of adipose tissue-derived molecules to the circulation and drives the development of obesity-derived metabolic complications namely insulin resistance, cardiovascular disease, and non-alcoholic fatty liver disease [18]. The association between the increased circulatory levels of LMW HA molecules and obesity have been poorly addressed and few studies evidenced a close relationship between insulin resistance and serum and tissue accumulation of HA in both type 2 [3, 19–21] and type 1 diabetic patients, the latter due to important pancreatic tissue damage [22]. In the current study, we were aimed at investigate if obesity, which entails an adipose tissue deregulated expansion, leads to a plasma increase of the LMW HA molecules which alternatively could act as damage-associated molecular patterns (DAMPs), triggering pro-inflammatory responses in human leukocytes and contributing to the development of low-grade inflammation in individuals with obesity.

## Materials and methods

### Study participants and sample collection

A total of 37 individuals undergoing laparoscopic surgery were recruited from the Gastroenterology Surgery Unit of either the Hospital Clínic of Barcelona or the Hospital Mútua de Terrassa. Demographic and clinical data were collected from patients’ electronic medical records at the time of surgery. Individuals with inflammatory bowel disease or cancer and obese patients with previous bariatric surgery were excluded from the study. From each subject, 10 ml of whole blood were collected prior to surgery and a sample of omental adipose tissue was obtained at the moment of the procedure for subsequent experiments. Briefly, tissue harvested samples were washed twice with DPBS and minced into 60 mg pieces, snap-frozen in liquid nitrogen and stored at −80 °C for further analysis. Additionally, 10 ml of whole blood were collected from healthy non-obese volunteers (*n* = 14) at the Banc de Sang i Teixits of the Hospital Clínic of Barcelona and were included in the study. Participants were selected according to their BMI calculated as mass/(height)^2^ and categorized as control (BMI < 29.9 kg/m^2^, *n* = 18) and obese (BMI > 29.9 kg/m^2^, *n* = 33). All studies were conducted in accordance with the criteria of the Investigation and Ethics Committee from either the Hospital Clínic and the Hospital Mútua de Terrassa and written informed consent was obtained from all participants.

### Plasma LMW HA isolation by ion-exchange chromatography

Separation of plasma HA content according to molecular mass was performed by ion-exchange chromatography using an adapted method described by Yuan et al. [23] (Supplementary Fig. S1B). Fractionation was assessed by gel electrophoresis using an adaptation of the method described by Bhilocha et al. [24]. Further details included as Supplementary Information.

### Measurement of HA levels

Levels of HA from both total, and 0.425 M NaCl plasma eluted fractions were determined using the Quantikine^®^ ELISA Hyaluronan Immunoassay (R&D Systems Inc., MN, USA) according manufacturer’s instructions. This is a specific quantitative sandwich enzyme immunoassay designed to measure ≥35 kDa HA in human samples. Further details included as Supplementary Information.

### Human leukocyte isolation and cell incubations

Peripheral blood monocytes (PBMC) and neutrophils (PMN) were isolated from 10 ml of whole blood by the Ficoll-Hypaque method. Human leukocytes and the human monocyte cell line THP-1 (ATCC, Manassas, VA,USA) were cultured at a density of 1–2 × 10^6^ cells/mL in RPMI 1640 medium containing 10% FBS, L-glutamine and antibiotics at 37 °C in a 5% CO_2_ incubator. Cell incubations were performed in the presence of either vehicle (0.01% sterile H_2_O), 100 ng/ml LPS (Sigma Aldrich, St Louis, MO, USA) or increasing concentrations of LMW/IMW HA (50, 100, 150 and 200 μg/mL) (R&D Systems Inc., MN, USA) for 6 h in a humidified 5% CO_2_ incubator at 37 °C. For the assessment of NFκB intracellular signaling, PBMC, and THP1 cells were incubated in the presence of either vehicle (0.01% EtOH) or 10 μM of the proteasome inhibitor MG132 (Merck Millipore, MA, USA) for 30 min before the addition of either LMW HA (100 μg/ml and 100 pg/ml) or 100 ng/ml of the Toll-like receptor 2 (TLR2) agonist Pam2CSK4 (InvivoGen, CA, USA) for 2 and 6 h. At the end of the incubation periods, cells were washed and collected for further gene expression analysis. Further details included as Supplementary Information.

### Human Adipose tissue explants and ex vivo incubations

Human visceral adipose tissue from patients with obesity was collected under sterile conditions. Treatments were performed in DMEM with L-glutamine, antibiotics and 1% endotoxin-free BSA–fatty acid-free (FAF) (Sigma Aldrich, St Louis, MO, USA). Briefly, tissue explants were incubated with vehicle (0.01% EtOH) and the recombinant cytokines IL-1β (25 pg/ml), IL-6 (10 ng/ml) and IL-10 (20 ng/ml) (R&D Systems Inc., MN, USA) for 6 h at 37 °C. At the end of the incubation period, tissue explants were frozen at −80 °C for further mRNA expression analysis of HAS1. Further details included as Supplementary Information.

### RNA isolation, reverse transcription and real-time PCR

Isolation of total RNA from adipose tissue, PBMC, PMN and THP-1 cells was performed using the TRIzol reagent (ThermoFisher Scientific, Waltham, MA, USA) and real-time PCR analysis was performed in the 7900HT Fast System (Applied Biosystems, MA, USA). Further details included as Supplementary Information.

### Western blot analysis

Total protein was separated by SDS-PAGE and transferred onto PVDF membranes by the iBlot Dry Blotting System (Invitrogen, MA, USA). Blots were incubated overnight at 4 °C with primary anti-human antibodies for phospho-IKK α/β (2697 S, 1:1000; Cell Signaling Technology, MA, USA), IKK α/β (sc-7607,1:400; Santa Cruz Biotechnology, TX, USA) phospho-IKB-α (9246 S, 1:200; Cell Signaling Technology) and IKB-α (sc-203, 1:200; Santa Cruz Biotechnology). Thereafter, the blots were incubated with either donkey anti-rabbit (Biolegend, CA, USA) or anti-mouse (Cell Signaling Technology) HRP-linked antibody (1:2000). To assess housekeeping protein expression, membranes were reblotted overnight at 4 °C with primary rabbit anti-human β-actin HRP conjugate (Cell Signaling Technology). Bands were visualized using the EZ-ECL chemiluminescence detection kit (Biological Industries, Israel). Total IKK α/β, IKB-α and β-actin were used as internal controls when applicable to verify basal level expression and equal protein loading. Further details included as Supplementary Information.

### Luminex xMAP technology

Cytokine levels were determined in plasma samples using a Milliplex MAP Human Cytokine/Chemokine Magnetic Bead Panel (Merck Millipore, MA, USA) on a Luminex 100 Bioanalyzer (Merck Millipore). Further details included as Supplementary Information.

Statistical analysis of the results was performed by analysis of variance for multiple comparisons (one-way or two-way ANOVA) or the unpaired Student’s *t* test for single comparisons. An adjusted *p*-value ≤ 0.05 was considered statistically significant.

## Results

### Patient characteristics and markers of systemic inflammation

**Fig. 1:**
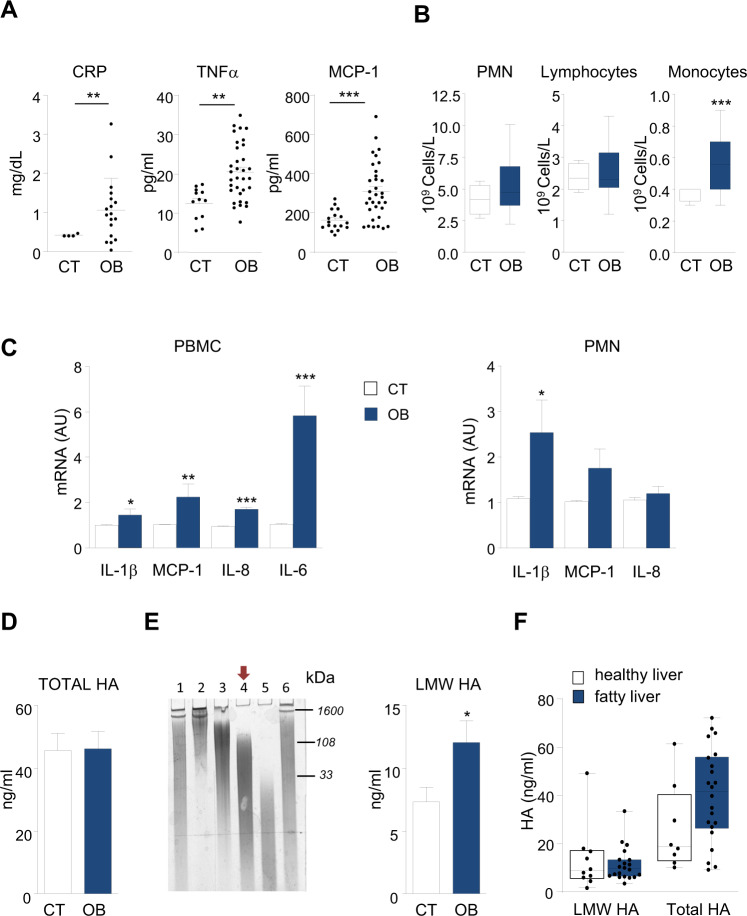
Systemic low-grade inflammation, PBMC activation, and increased plasma levels of LMW HA in obese patients. **A** Plasma levels of C-reactive protein (CRP), TNFα, and MCP-1 in control participants (CT) and individuals with obesity (OB). **B** Total blood cell count for neutrophils (PMN), lymphocytes, and monocytes in control (CT) and obese (OB) patients. **C** IL-1β, MCP-1, IL-8, and IL-6 mRNA expression in freshly isolated PBMC and PMN from control participants (open bars) and individuals with obesity (solid bars). **D** Total levels of plasma hyaluronic acid (HA) were directly assessed by enzyme-linked immunoassay in control participants (CT, *n* = 27) and individuals with obesity (OB, *n* = 33). **E** *Left panel*, representative gel image of a HA Ladder sample fractionation analysis by electrophoresis. Briefly, HA Ladder was prepared by mixing three commercial polydisperse hyaluronan polymers produced by microbial fermentation of *Streptococcus pyogenes* (Fig. S1A and Supplementary Information): HMW, IMW and LMW HA. Their weight-average MW corresponding to 1600 kDa (HMW HA), 108 kDa (IMW), and 33 kDa (LMW) HA is shown on the right of the gel image. The resulting eluted size-specific HA fractions were co-electrophoresed along with the HA Ladder sample to assess the size distribution according to the fractionation procedure. *Line 1*, polydisperse HA Ladder sample ranging from 15 to > 950 kDa; *line 2*, HMW HA fraction eluted from 0.800 M NaCl (>350 kDa); *line 3*, IMW HA fraction eluted from 0.460 M NaCl (75–350 kDa); *line 4*, LMW HA fraction eluted from 0.425 M NaCl (15–40 kDa); *line 5*, very low MW HA eluted from 0.360 M NaCl and *line 6*, alternative HA Ladder sample made by combining equal volumes (5 μl) of the different stepwise eluted fractions showed in lines *2*, *3*, *4*, and *5*. *Right panel*, plasma levels of LMW HA were quantified in 0.425 M NaCl eluted fraction from control participants (CT, *n* = 13) and individuals with obesity (OB, *n* = 30) by enzyme-linked immunoassay. **F** Total and LMW HA levels in patients with obesity categorized in two groups by the presence (solid bars) or absence (open bars) of hepatic steatosis assessed by ultrasound examination. Data are mean ± SEM. *p < 0.05, **p < 0.005, ***p < 0.0005 versus CT subjects.

Individuals included in the study were categorized according to their body mass index (BMI, Kg/m^2^) in non-obese (CT, BMI < 29.9) and obese (OB, BMI > 29.9) groups. Anthropometric and biochemical parameters collected prior to laparoscopic surgery are shown in Table 1. Elevated plasma levels of C-reactive protein (CRP) and pro-inflammatory cytokines such as IL-6, TNF-α, MCP-1, IL-8, and IL-7 confirmed the existence of an inflammatory state in individuals with obesity (Fig. 1A and Table 2). Importantly, a strong positive association was found between BMI and circulating levels of most significantly differentiated pro-inflammatory cytokines namely IL-6, MCP-1, IL-8, and the anti-inflammatory cytokines IL-1ra and IL-10 (Supplementary Table 2). Systemic inflammation was associated with increased numbers of circulating monocytes (Fig. 1B) and marked signs of leukocyte activation as shown in Fig. 1C. Mostly in freshly isolated PBMC from individuals with obesity which showed significantly higher expression levels of pro-inflammatory cytokines (IL-1β, MCP-1, IL-8, and IL-6) as compared to control subjects (Fig. 1C). PMN from OB exhibited up-regulated levels of IL-1β with no changes on MCP-1 and IL-8 when compared to CT.

**Table 1:** Clinical characteristics and anthropometric measurements of the study participants.

	CT (*n* = 18)	OB (*n* = 33)
Gender		
Male	8	11
Female	10	22
Age (years)	46.4 ± 1.7	43.0 ± 1.7
BMI (kg/m^2^)	19.6 ± 0.5	42.6 ± 0.7^a^
Steatosis (%)	–	18/33 (70%)
Glucose (mg/dL)	87.0 ± 3.2	105.5 ± 4.4^b^
Glycated Hemoglobin (%)	–	5.9 ± 0.1
Triglycerides (mg/dL)	108 ± 25.3	132 ± 12.3
Cholesterol (mg/dL)	176 ± 7.7	188 ± 6.7
ALT (UI/L)	17.2 ± 3.3	31.9 ± 2.8
AST (UI/L)	32.4 ± 7.1	24.2 ± 1.7
Albumin (g/L)	38.3 ± 1.4	42.6 ± 0.5^b^
Platelets (10^9^/L)	300 ± 29.7	288 ± 12.2

CT: non-obese individuals (BMI < 29.9), OB: individuals with obesity (BMI > 29.9).

Normal reference index: glucose: 65–110 mg/dL, glycated hemoglobin: 4–6%, triglycerides: 50–150 mg/dL, cholesterol: 148–247 mg/dL, albumin: 34–48 g/L, platelets: 150–400 10^9^/L, ALT and AST: 5–40 UI/L. Mean ± SEM.

*BMI* body mass index, *ALT* alanine aminotransferase, *AST* aspartate aminotransferase.

^a^*P* < 0.0001.

^b^*P* < 0.05 vs CT.

**Table 2:** Plasma levels of cytokines in control participants (CT, BMI < 29.9) and individuals with obesity (OB, BMI > 29.9) as analyzed by multiplex xMAP technology.

	CT (*n* = 18)		OB (*n* = 33)		
Cytokine	Mean	SEM	Mean	SEM	*P* value
IL-6	0.5	0.1	2.9	0.4	0.0001
IP-10	103.1	9.9	176.2	14.0	0.0001
IL-1ra	6.1	1.1	33.2	5.5	0.0001
MCP-1	180.5	22.2	308.2	25.4	0.0005
TNF*α*	12.5	1.2	20.6	1.3	0.001
IL-8	2.5	0.4	4.4	0.5	0.005
IL-10	1.1	0.2	3.0	0.4	0.005
IL-7	3.3	0.4	5.7	0.8	0.01
MIP-1*β*	15.3	1.6	20.6	1.4	0.05
VEGF	75.1	12.2	113.7	13.9	0.05
G-CSF	6.4	1.5	14.0	2.5	n.s.
IFN*γ*	9.1	1.7	12.1	1.5	n.s.
IFN*α*2	42.0	4.8	49.2	4.5	n.s.
Eotaxin	37.5	4.3	40.8	3.0	n.s.
IL-17A	3.7	0.5	5.2	0.5	n.s.
MIP-1*α*	16.3	2.3	18.6	1.3	n.s.
IL-1*α*	4.6	0.9	6.2	0.7	n.s.
IL-1*β*	6.5	1.1	5.3	0.6	n.s.
IL-4	1.4	0.2	1.6	0.3	n.s.

Results are expressed as pg/ml of plasma and as mean ± SEM.

*BMI* body mass index.

### Increased plasma levels of LMW HA in obese patients

To study the role of circulating HA as a potential self-danger/alarm signal for leukocyte activation in obesity, we first measured the total HA plasma levels in CT and OB individuals. Although we found a strong correlation between serum glucose levels and total HA levels in plasma (*r* = 0.54, *P* < 0.001; *n* = 35 XY pairs, Spearman’s rho test), the total circulatory content of HA was not significantly different between CT and OB individuals (Fig. 1D). As HA is found to be a size-dependent modulator of inflammation we performed a previously described method [23] to specifically measure plasma content in LMW HA fragments. To test this methodological approach, we used commercially available specific-size HA molecules each one containing a polydisperse mixture of HA fragments with a particular MW average, namely, high MW HA (HMW HA, 950 KDa), Intermediate MW (IMW HA, 108 KDa) and LMW HA (33 KDa). The specific-size pattern for each polydisperse HA molecules is shown in Supplementary Fig. S1A. As shown in Fig. 1E, *left panel*, the size assessment of each HA stepwise eluted fraction (*lanes 2*, *3*, *4*, and *5*) and both total HA Ladder prior to fractionation (*lane 1*) and HA resulting from combined eluted fractions (*lane 6*) confirmed a precise ion-exchange column fractionation giving rise in each eluted solutions a polydisperse smear of HA molecules according to their expected size pattern. Once we established the procedure, and as commented before, human plasma samples from CT and OB individuals were fractionated along with a HA Ladder, and fraction eluted from 0.425 M NaCl, corresponding to LMW fragments of HA, was then quantified by ELISA. As shown in Fig. 1E, *right panel*, plasma from OB showed significantly higher levels (ng/ml) of LMW HA than CT (CT, 7.3 ± 1.2, *n* = 13 vs OB, 12.0 ± 1.7, *n* = 30, *P* < 0.05). Importantly, among a panel of 19 cytokines, LMW HA levels were found to be closely associated with both pro-inflammatory interleukins IL-8 (*r* = 0.38, *P* < 0.05; 42 XY pairs, Spearman’s rho test) and IL-7 (*r* = 0.42, *P* < 0.01, 42 XY pairs, Spearman’s rho test). As the presence of important structural liver tissue changes is seen in people with obesity, we then analyzed HA levels according to the presence or absence of liver steatosis in individuals with obesity. Although a slight increase in total HA levels was observed in OB patients presenting fatty liver, no significant statistical differences were found for neither total nor LMW HA in comparison with OB patients with an ultrasound image of healthy liver (Fig. 1F).

### Size-dependent inflammatory effects of HA on healthy leukocytes

**Fig. 2:**
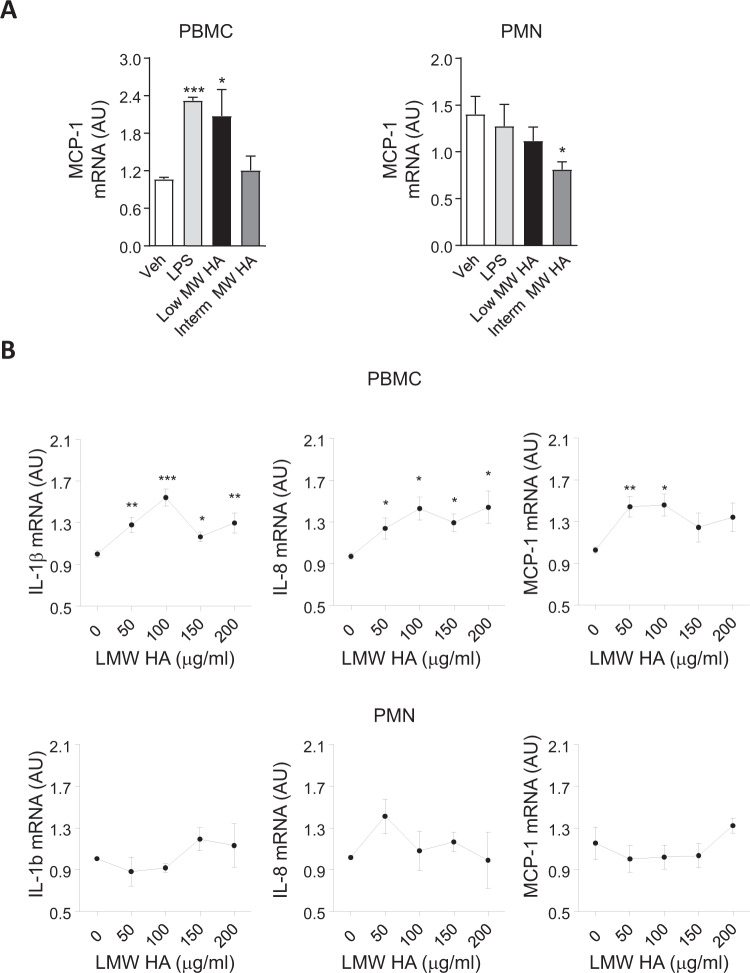
Low and Intermediate molecular weight HA effects on cytokine expression in both healthy PBMC and PMN. **A** Relative mRNA levels for MCP-1 in both healthy human PBMC and PMN incubated for 6 h in the absence (Veh) or presence of LPS (100 ng/ml), low MW HA (100 μg/ml), and Intermediate MW HA (100 μg/ml). **B** Relative mRNA levels for IL-1β, IL-8, and MCP-1 in both healthy human PBMC and PMN incubated for 6 h in the presence of increasing concentrations of LMW HA (0, 50, 100, 150, and 200 μg/mL). Data are mean ± SEM of *n* = 3 independent experiments performed in duplicate. *p < 0.05, **p < 0.005, ***p < 0.0005 versus vehicle.

We next investigated the ability of different size HA molecules to induce a pro-inflammatory response in PBMC and PMN isolated from healthy volunteers. As shown in Fig. 2A, PBMC display up-regulated MCP-1 expression in response to LMW HA in a similar extent to those incubated with LPS whereas no changes were observed upon PBMC challenged to IMW HA (Fig. 2A and Supplementary Fig. S2). Dose-dependent experiments on cultured leukocytes confirmed that LMW HA at concentrations ranging from 50 μg/ml to 200 μg/ml produced a significant stimulatory effect on cytokine expression in PBMC (Fig. 2B). Of note, freshly isolated PMN were not responding to LPS either to low or intermediate size HA and only a significant anti-inflammatory effect was observed for IMW HA in freshly isolated PMN a finding that supports opposite roles for HA according to its molecular size (Fig. 2 and Supplementary Fig. S2).

### Obesity modulates the expression of HA receptors in leukocytes and alters their pro-inflammatory response to LMW-HA

**Fig. 3:**
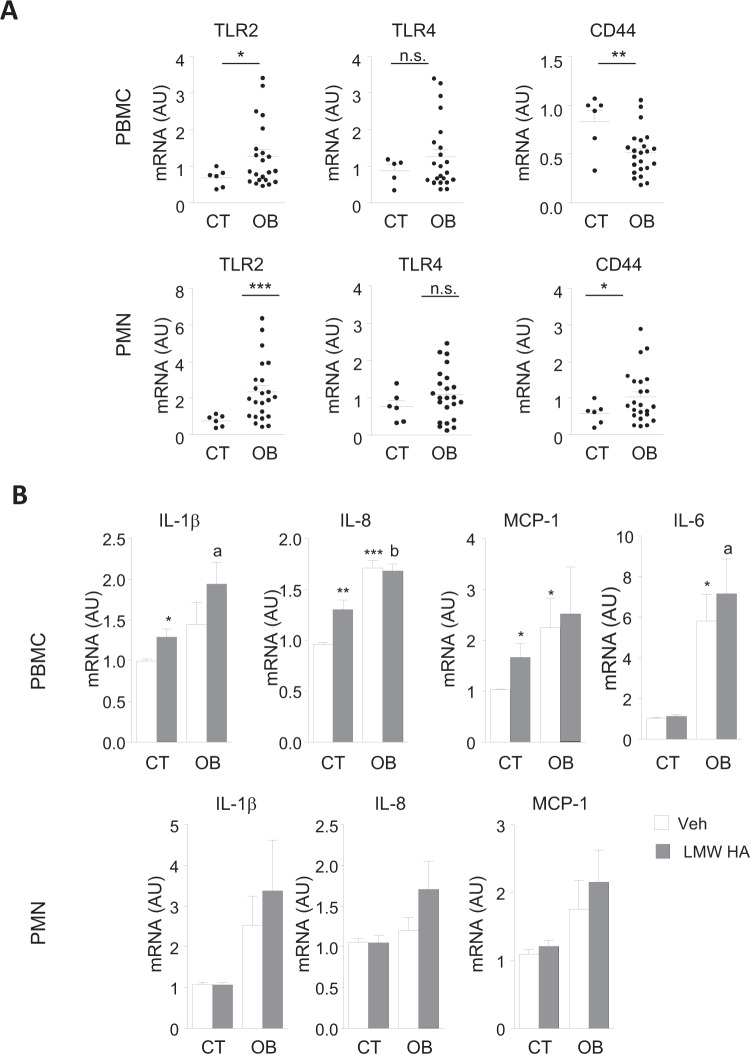
Pro-inflammatory effects of LMW HA on healthy and not on obese derived PBMCs in vitro. **A** TLR2, TLR4, and CD44 mRNA expression in freshly isolated PBMC and PMN from control participants (open bars) and (solid bars) individuals with obesity. **B** Relative mRNA levels for IL-1β, IL-8, IL-6, and MCP-1 in both healthy human PBMC and PMN incubated for 6 h in the presence of vehicle (sterile water) or LMW HA (100 μg/mL), IL-6 mRNA levels were undetectable in PMN. Data are mean ± SEM *n* = 3 independent experiments performed in duplicate. *p < 0.05, **p < 0.005, ***p < 0.0005 versus CT subjects or CT-vehicle-treated cells, and ^a^*p* < 0.05, ^b^*p* < 0.005 versus CT LMW HA-treated cells.

The expression of the canonical CD44 receptor and non-canonical TLR2 and TLR4 for HA was next examined on freshly isolated leukocytes from CT and OB individuals. As shown in Fig. 3A, as compared to CT, OB showed a marked up-regulation of TLR2 mRNA levels in either PBMC and PMN and an opposite gene expression modulation for CD44 between PBMC and PMN being down-regulated in obese PBMC whereas TLR4 did not change. A deeper analysis on the in vitro response of cultured leukocytes from CT and OB to a pro-inflammatory dose of LMW HA revealed that only PBMC from non-obese were able to respond significantly to LMW HA by increasing the expression of IL-1β, IL-8, and MCP-1. PBMC from OB remained strongly activated in culture, expressing higher levels of pro-inflammatory cytokines than CT leukocytes and, importantly, no further additive effects on this pro-inflammatory state were observed upon LMW HA challenge (Fig. 3B, *upper panel*). Moreover, LMW HA did not change cytokine expression either in CT or OB PMN pointing out blood PBMC as a cell target for pro-inflammatory effects of LMW HA.

### LMW HA upregulates pro-inflammatory markers through NF-κB-dependent signaling in PBMCs and THP-1 monocytes

**Fig. 4:**
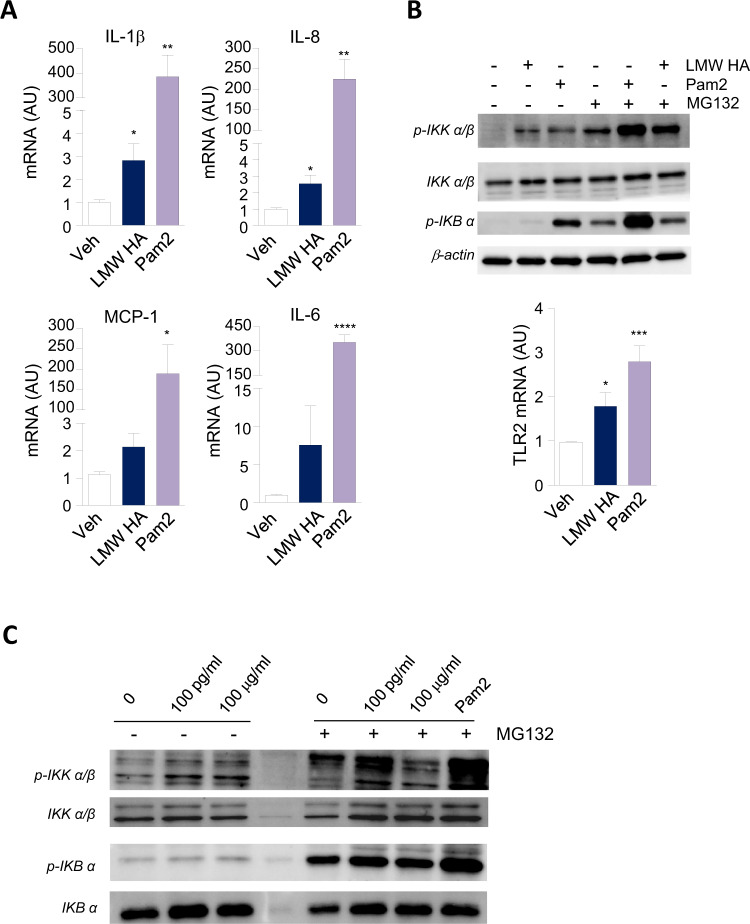
LMW HA upregulates pro-inflammatory markers through NF-κB-dependent signaling in PBMCs and THP-1 monocytes. **A** Relative mRNA levels for IL-1β, IL-8, MCP-1, and IL-6 in human monocyte-like THP-1 cells incubated for 6 h in the presence of vehicle (sterile water), LMW HA (100 μg/mL), and Toll-like receptor 2 (TLR2) agonist Pam2CSK4 (100 ng/ml). **B** *Upper panel*, Representative immunoblots of IKK α/β, pIKK α/β, pIKB α and β-actin in THP-1 cells incubated with vehicle (0.01% ethanol) or MG132 (10 mM) for 30 min before the addition of vehicle (sterile water), Pam2CSK4 (100 ng/ml) or LMW HA (100 μg/mL) (10 ng/ml) for 2 h. *Lower panel*, relative mRNA levels for TLR2 in human monocyte-like THP-1 cells incubated for 6 h in the presence of vehicle (sterile water), LMW HA (100 μg/mL), and Toll-like receptor 2 (TLR2) agonist Pam2CSK4 (100 ng/ml). Data are mean ± SEM *n* = 3 independent experiments performed in duplicate. *p < 0.05, ***p < 0.0005 versus vehicle-treated cells. **C** Representative immunoblots of IKK α/β, pIKK α/β, pIKB α and ΙΚΒ *α* in human healthy PBMC incubated with vehicle (0.01% ethanol) or MG132 (10 mM) for 30 min before the addition of vehicle (sterile water), increasing doses of LMW HA (100 pg/ml, and 100 μg/ml), and Pam2CSK4 (100 ng/ml) for 2 h. Results are expressed as mean ± SEM of 3 independent experiments performed in duplicate. *p < 0.05, **p < 0.005, ***p < 0.0005 versus vehicle.

To explore the intracellular mechanism of LMW HA in leukocyte response, we first assessed whether LMW HA activates canonical NF-κB signaling pathway in the human monocyte-like THP-1 cells. We confirmed that mRNA levels of NF-κB downstream target genes were significantly up-regulated by LMW HA and the specific TLR2 agonist, Pam2CSK4 (Fig. 4A) in cell incubations. Of note, gene expression levels of TLR2 were found to be also stimulated by LMW HA (Fig. 4B, *lower pane*l). As expected, the addition of the positive control, Pam2CSK4, induced the phosphorylation of IκB kinase (IKKα/β) which later on phosphorylates IKBα resulting in its proteasome-mediated degradation and subsequent activation of NF-κB-dependent gene transcription of IL-1β, IL-8, MCP-1 and IL-6 (Fig. 4A, B). The addition of LMW HA showed a similar level of IKKα/β phosphorylation than that observed for Pam2CSK4 but a quick and transient activation IKBα phosphorylation as confirmed upon blockade of proteasome degradation by pre-incubating THP-1 cells with MG132 (Fig. 4B, *upper panel*). The examination of this pathway on human leukocytes revealed that low concentrations of LMW HA as 100 pg/ml were able to activate NF-κB signaling in cultured healthy PBMC, result that supports the role of LMW HA as an inducer of TLR-derived pro-inflammatory response in obesity (Fig. 4C).

### Obese adipose tissue overexpresses hyaluronic acid synthase-1 (HAS-1) and IL-1β induces it expression

**Fig. 5:**
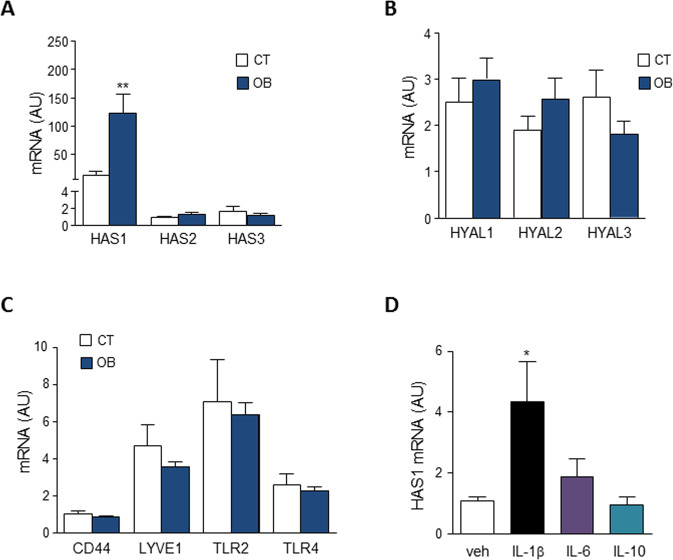
Obese adipose tissue overexpresses hyaluronic acid synthase-1 (HAS1) and IL-1β induces it expression. **A** HAS1, HAS2, and HAS3 mRNA expression in human visceral adipose tissue from control (CT, *n* = 5, open bars) and obese (OB, *n* = 18, solid bars) patients. **B** HYAL1, HYAL2, and HYAL3 mRNA expression in human visceral adipose tissue from control (CT, *n* = 5, open bars), and obese (OB, *n* = 18, solid bars) patients. **C** Relative mRNA levels for HA canonical receptors (CD44, LYVE1) and TLR2 and TLR4 in human visceral adipose tissue from control participants (CT, *n* = 5, open bars) and individuals with obesity (OB, *n* = 18, solid bars). **D** Changes in HAS1 gene expression levels in cultured human OB adipose tissue explants incubated in the presence of IL-1β (25 pg/ml), IL-6 (10 ng/ml) and IL-10 (20 ng/m) for 6 h. Results are expressed as mean ± SEM. **p < 0.005 versus CT subjects; ^a^*p* < 0.05 versus vehicle-treated explants in 3 independent experiments performed in duplicate.

We next investigated the gene expression status of ECM components implicated in HA turnover namely, hyaluronan synthases (HAS1, HAS2, and HAS3) and hyaluronidases (HYAL1, HYAL2, HYAL3) in human adipose tissue from both groups of study (CT and OB). As shown in Fig. 5A, HAS1 was found to be highly overexpressed in adipose tissue from obese patients and no changes were observed for the synthetic enzymes HAS2 and HAS3 or the HA degradative counterparts HYAL1, HYAL2, HYAL3 (Fig. 5B). Moreover, gene expression of surface receptors involved in HA actions was no modulated by obesity in adipose tissue (Fig. 5C). We further examine HAS1 transcriptional regulation by incubating cultured human adipose tissue explants with recombinant human cytokines implicated in low-grade inflammation, namely IL-1β, IL-6, and IL-10. Importantly, only pro-inflammatory IL-1β was able to up-regulate HAS1 gene expression in cultured human adipose tissue explants (Fig. 5D).

## Discussion

Increased blood levels of HA have been shown to correlate with metabolic comorbidities associated with obesity namely liver fibrosis raised from non-alcoholic hepatic steatosis [2], atherosclerosis [25], and diabetes [3]. Most of these published data refer to total HA content based on direct quantification of serum levels by conventional enzyme immunoassay which not allows size discrimination of HA extracellular fragments [26]. In the present study, we have adapted a previously described method [23] that uses anion exchange spin columns to size-dependent fractionation of plasma HA content and subsequent measurement of purified LMW HA molecules by conventional HA immunoassay. Importantly, we demonstrated for the first time that patients with obesity show increased circulating levels of LMW HA fragments whereas the total content of plasma HA molecules remains unchanged between control participants and individuals with obesity. This result led us to investigate visceral adipose tissue as the primary source of circulating HA small molecules in obesity. In this regard, and in murine models of inflammation, the accumulation of LMW HA has been ascribed to both the presence of an altered HA enzymatic metabolism [27] and increased non-enzymatic degradation by reactive oxygen species (ROS) which are able to promote tissue HA depolymerization to small fragments [17]. Our results were compatible with the presence of a combination of these two deregulated biological mechanisms occurring simultaneously in obese visceral adipose tissue. Regarding HA metabolic turnover, the statistically significant up-regulation of HAS1 in individuals with obesity with no relevant gene expression changes in HAS2 and HAS3 or HA degrading enzymes was confirmed in our previous microarray public data. Importantly, HAS1 transcript levels were found to be 14.34-fold change up-regulated (*P* = 0.0024) in obese adipose tissue being the highest altered gene expression observed among all significantly differentially expressed genes between control participants and individuals with obesity (Gene Expression Omnibus under accession number GSE71415). Although published current data are limited, recent studies have emphasized the role of HAS1 in metabolic inflammation as its activity is induced by high glucose levels and cytokines to produce a special type of pericellular hyaluronan coat with pro-inflammatory properties [28, 29]. In this context, pre-clinical studies have demonstrated that the accumulation of HA occurs in muscle from obese mice contributing to the development of insulin resistance and that therapeutic approaches using hyaluronidases or HA synthesis inhibitors show improvements in metabolic outcomes and reduce adipose tissue hypertrophy and inflammation [20, 30]. Our results indicating that HAS1 gene expression could be up-regulated by pro-inflammatory cytokine IL-1β, and that obese patients show both elevated levels of HAS1 mRNA in adipose tissue and plasmatic LMW HA supports the notion that increased hyaluronan synthesis is close-related with the inflammatory state in obesity. Despite this solid evidence, we cannot exclude the non-enzymatic generation of small fragments of HA through ROS-mediated direct cleavage since free oxygen radicals are overproduced during fat mass expansion and could contribute to rising up the LMW HA levels in plasma [17, 31] a subject that we have not addressed in this study and requires further investigation.

Besides adipose tissue, the liver is another organ usually suffering from ECM disturbances in obese patients and might be also contributing to the plasmatic LMW HA content [32]. Fat accumulation in hepatocytes activates a process of ECM remodeling leading to obese-derived non-alcoholic liver disease newly better defined as metabolic-associated fatty liver disease (MAFLD) [33]. In our study 70% of obese patients showed hepatic steatosis after the ultrasound examination nevertheless, our findings indicated that increased LMW HA levels were independent of the presence or absence of hepatic steatosis pointing to adipose tissue as a primary source of LMW HA.

It is well known that obese visceral adipose tissue plays a major role in the systemic inflammatory state through the unbalance release of the so-called adipokines but also free fatty acids and DAMPs into circulation altering immune homeostasis in individuals with obesity [34]. Our findings confirm a state of systemic low-grade inflammation in obese patients as they presented higher circulating monocyte count and increased plasma levels of classical molecules involved in inflammation namely interleukins, chemokines, the growth factor VEGF, and TNFα. At a cellular level, we found that obesity elicits an activated pro-inflammatory state in both freshly isolated PBMC and PMN. This leukocyte behavior has also been observed in previous publications [35] and confirms that blood leukocytes are also contributing to the overall state of chronic low-grade inflammation in individuals with obesity.

Our results on the impact of HA molecules on blood leukocytes, support the general notion that the functional role of HA fragments in the inflammatory response is dependent on their molecular size [32, 36–38]. We confirmed previous findings indicating that small fragments of HA exert pro-inflammatory effects on leukocytes [39, 40]. Specifically, we found that PBMC were particularly reactive to increasing concentrations of LMW HA molecules whereas PMNs remained un-reactive neither to LPS nor LMW HA. Only a significant anti-inflammatory effect was observed when PMN were stimulated with IMW HA a finding that supports described opposite biological actions for HA molecular species of different size [8]. On this subject, the general consensus ascribes anti-inflammatory properties to HMW HA molecules ranging > 10^6^ kDa and pro-inflammatory actions to those HA polymers < 500 kDa [10, 11]. Our data regarding this distinct size-dependent effect of molecules < 500 kDa are in line with other published studies that reveal that HA could exert its biological actions through more complex mechanisms than by a mere matter of size. In fact, HA actions depend on factors as the cell type, the pathophysiological context either in fluids or in solid tissues and importantly, the HA receptors involved, their clustering, and their interaction with other cell surface molecules as selectins [11, 26, 41, 42]. The relevant range of concentrations of LMW HA affecting the behavior of blood leukocytes in vivo is another aspect that deserves further investigation. In our in vitro experiments, high doses of LMW HA (50–200 μg/ml) have shown functional relevance while lower doses as 100 pg/ml only have demonstrated NFκB activation without evidence of gene expression changes (data not shown). One of the limitations of our study is that the in vitro model of leukocyte assays is not capturing all the microenvironmental aspects of what is occurring in vivo. Thus, no changes in cytokine gene expression were observed using lower concentrations of LMW HA.

As we have shown, canonical HA receptor (CD44), and TLRs 2 and 4, were found to be expressed by PBMC and PMN in our cohort of individuals. Higher levels of CD44 in obese PMN might be in line with previously published data showing that HA-CD44 interaction is the main mechanism for leukocyte trafficking from the bloodstream into inflamed tissues [43] and PMN recruitment within the hepatic microvasculature in systemic inflammation [44]. In fact, CD44 has found to play a key role in non-alcoholic steatohepatitis development, a hepatic complication of MAFLD closely associated to the presence of obesity [45]. Nevertheless, knowing the precise effects of HA fragments on blood leukocyte behavior through their interaction with CD44 are at present unknown and need further studies in the setting of obesity. On the other hand, it has been shown that both TLRs 2 and 4 play crucial roles in the inflammatory response to HA fragments [13, 15, 46]. We have observed that obesity induced a remarkable increase in TLR2 gene expression in both PBMC and PMN while TLR4 mRNA levels remained unchanged. Dasu et al. previously described that TLR2 and TLR4 were increased in monocytes from type 2 diabetic patients and importantly, that HA molecules as well as some of other tissue-damaged associated molecules (HMGB1 and HSP61), co-immunoprecipitated with these receptors and might be linked to their activation through the TLR/NF-κB-dependent pathway [19]. Numerous studies have evidenced that harmful endogenous molecules of non-infectious origin could trigger a state of inflammation by the activation of these innate immune receptors [47]. In agreement with a nice study from Scheibner et al. [46] focused on murine cells, we have demonstrated that LMW HA activate NF-κB-dependent pathway in human PBMC and THP-1 cells thus acting as a danger alarm molecule and suggesting an important role in TLRs activation and its contribution to low-grade inflammation. Interestingly, LMW HA did not induce a further pro-inflammatory effect on obese leukocytes suggesting that small molecules of HA exert its pro-inflammatory effects early in the progression of obesity.

Summarizing, we demonstrated for the first time that patients with obesity show increased circulating levels of LMW HA fragments in plasma. We also provided additional evidence supporting the role of these small fragments of HA (15–40 kDa) as DAMP molecules in blood leukocytes. Specifically, by inducing the expression of pro-inflammatory genes through TLR/NF-κB signaling in PBMC, LMW HA could be an early contributor to the development of low-grade state of inflammation, the prelude of further metabolic complications associated to obesity.

## Supplementary information

Supplementary Information

## Data Availability

The data that support the findings of this study are available from the corresponding author, ET, upon reasonable request.
