# Supplementary material for: Small fragments of hyaluronan are increased in individuals with obesity and contribute to low-grade inflammation through TLR-mediated activation of innate immune cells
Source: Int J Obes (Lond). 2022 Jul 27;46(11):1960–9. doi: 10.1038/s41366-022-01187-z (PMC9584819; doi:10.1038/s41366-022-01187-z)
Supplement: Supplementary file 1 — Supplementary Information [file 41366_2022_1187_MOESM1_ESM.pdf]

Supplementary Information for

Small Fragments of Hyaluronan are Increased in Individuals with Obesity and Contribute to Low-Grade Inflammation through TLR-mediated activation of Innate Immune Cells by Romo M et al.

The pdf file includes:

Materials and Methods.

Supplementary TABLE 1.

Figure S1. Plasma LMW HA isolation by IEX chromatography.

Figure S2. Intermediate MW HA effects on cytokine expression in leukocytes.

MATERIALS AND METHODS

Biochemical analyses: Plasma concentrations of glucose, cholesterol, TAG, ALT and AST were determined by standard laboratory procedures.

Plasma LMW HA isolation by ion-exchange chromatography

Sample preparation

Separation of plasma HA content according to molecular mass was performed by ion-exchange chromatography using an adapted method described by Yuan H *et al.* (23) (Supplemental Fig. S1B). Before chromatographic purification, plasma samples (700 µl) were centrifuged for 10 minutes at 1500 g and digested using 0.5 mg/mL of proteinase K (Roche Diagnostics, Basel, Switzerland) overnight at 60ºC with agitation at 900 rpm. Samples were then placed on ice for 10 min and centrifuged at 16000 g for 40 min at 4ºC to separate the aqueous content from the lipid

layer. Cleared plasma was collected and submitted to chromatography along with a synthetic polydisperse HA mixture (HA Ladder) of known concentration to monitor plasma HA fractionation by gel polyacrylamide electrophoresis as described below.

Polydisperse HA mixture preparation (HA Ladder)

To monitor HA fractionation, we prepared an aqueous HA standard mixture (HA Ladder) contained three commercial different-sized HA polymers ranging from 15 to > 950 kDa (R&D Systems, MN, USA). Briefly, High Molecular Weight (HMW) HA contained molecules > 950 kDa and the lot used certified a mass average of 1.67 x 10^6 Da (ref. GLR002, Lot. 1471247), Intermediate Molecular Weight (IMW) HA contained HA molecules within a molecular range of 75 to 350 kDa and had a mass average of 108 kDa (ref. GLR004, Lot. 1490159), and Low Molecular Weight (LMW) HA contained HA molecules ranging from 15 to 40 kDa and had a mass average of 33 kDa (ref. GLR001, Lot. 1510020). All three commercial polydisperse hyaluronan polymers were produced by microbial fermentation of *Streptococcus pyogenes* and contained less than 0.01 EU per mg of endotoxin as specified by the manufacturer in the corresponding certificate of analysis (R&D Systems Inc., MN, USA). The HA Ladder was prepared by mixing: 0.05 μg/μl of HMW HA, 0.2 μg/μl of IMW HA and 0.3 μg/μl LMWHA. This HA Ladder was submitted in parallel along with the plasma samples to the same fractionation procedure and HA levels prior and after fractionation were quantified by ELISA to measure the LMW HA recovery data that was calculated at 85 % for the Ladder LMW HA fraction.

Ion-exchange chromatography

Both cleared plasma and HA Ladder samples were dialyzed against 2 changes of deionized water using 3.5 kDa cut-off dialysis device (# 88403, Thermo Scientific, MA, USA) overnight. After the dialysis, samples were loaded onto strong anion exchange (Q) spin columns (#90010, Thermo

Scientific, MA, USA) for fractionation of HA polymers according to their MW using increasing concentrations of sodium chloride (NaCl) solutions. Briefly, each column was pre-washed with 400 µL of 0.05 M NaCl solution and then 400 µL of the dialyzed samples were loaded onto the spin columns, centrifuged at 2000 g for 2 min, and discarded the eluted solution. The spin columns were then washed once with 400 µL of 0.05 M NaCl followed by centrifugation at 2000 g for 5 min discarding the eluate. Additional three washes of 0.2 M NaCl were performed in the same manner. The specific sized-HA fractions were eluted from the spin columns by centrifugation at 2000 g for 2 minutes using 400 µl of increasing concentrations of NaCl as follows, 0.360 M (HA fragments below 15 kDa), 0.425 M (LMW HA, 15-40 kDa), 0.46 M (IMW HA, 75-350 kDa) and 0.8 M NaCl (HMW HA > 950 kDa). The eluted fractions were then loaded onto 3.5 kDa cut-off dialysis devices and were dialyzed again as described above. These dialyzed fractions were then transferred to 1.5 mL tubes and vacuum dried using the SpeedVac SPD1010 (Thermo Scientific, MA, USA). The dry residues were reconstituted in 50 µL of deionized sterile water, of them, 20 µl were used for the subsequent electrophoresis analysis and 25 µl of 0.460 M eluted fraction were used for ELISA quantitation of LMW HA levels (Supplemental Fig. S1B).

Fractionation assessment by gel- electrophoresis

Aliquots of 20 µl of eluted LMW HA fractions along with non-fractionated samples were electrophoresed for 1 h 40 min at 120 V using a 10% polyacrylamide gel in Tris-Borate-EDTA (TBE) buffer using an adaptation of the method described by Bhilocha S et al (24). The HA bands were visualized using 0.005% Stains-All™ dye (Sigma Aldrich, St Louis, MO, USA) in 50 % ethanol and images were obtained under visible light using a ImageQuant LAS4000 instrument (GE Healthcare Life Sciences, UK).

Measurement of HA levels

Levels of HA in diluted samples (1/4) from both total, and 0.425 M NaCl plasma eluted fractions were determined using the Quantikine® ELISA Hyaluronan Immunoassay (R&D Systems Inc., MN, USA) according manufacturer's instructions. This is a specific quantitative sandwich enzyme immunoassay designed to measure ≥ 35 kDa HA in human samples. Absorbance was obtained using a FLUOstar OPTIMA microplate reader (BMG Labtech, Ortenberg, Germany).

Human leukocyte isolation

Peripheral blood monocytes (PBMC) and neutrophils (PMN) were isolated from 10 ml of whole blood samples collected in ethylenediaminetetraacetic acid (EDTA) lined tubes. Firstly, and after centrifugation at 200 *g* for 10 min, plasma was collected and subsequently aliquoted and stored at -80ºC for further analysis. Sedimented cells were diluted with Dulbecco's Phosphate-Buffered Saline without calcium and magnesium ($DPBS^{--}$) (Lonza, Verviers, Belgium) up to a volume of 10 mL. Diluted blood cells were layered over 6.6 mL of Ficoll- Hypaque PLUS (GE Healthcare Life Sciences, MA, USA) and centrifuged at 500 g for 25 min with the break on. PBMC were obtained from the mononuclear cell layer, washed with $DPBS^{--}$ and centrifuged again at 400 g for 5 min. Pelleted PMN were incubated with pre-warmed ammonium-chloride-potassium lysis buffer (ACK) for 10 min at room temperature to remove red blood cells and then centrifuged at 400 g for 5 min. The red blood lysis procedure was repeated twice and the resultant pellet was washed with $DPBS^{--}$. Isolated viable PBMC and PMN were counted by Trypan Blue exclusion test and characterized by phase contrast microscopy using Diff-Quick staining. An aliquot of $4x10^6$ cells were immediately snap frozen for mRNA analysis and the remaining cells were then cultured overnight in a humidified 5% CO2 incubator at 37 ºC at a density of $2x10^6$ cells/mL in RPMI 1640 medium (Lonza, Verviers, Belgium) containing penicillin (100 U/mL)/treptomycin (100 U/mL) and L-glutamine (4 mM) with 10% fetal bovine serum (FBS).

Cell incubations

Human leukocytes and the human monocyte cell line THP-1 (ATCC, Manassas, VA ,USA) were cultured at a density of 1-2x10^6 cells/mL in RPMI 1640 medium containing 10% FBS, L-glutamine and antibiotics at 37ºC in a 5% CO_2 incubator. Briefly, freshly isolated leukocytes were maintained overnight in RPMI 1640 complete medium and then washed once with RPMI 1640 medium containing penicillin (100 U/mL)/streptomycin (100 U/mL) without FBS. Cells were subsequently incubated in the same RPMI 1640 medium in the presence of either vehicle (0.01% sterile H_2O), 100 ng/ml LPS (Sigma Aldrich, St Louis, MO, USA) or increasing concentrations of LMW/IMW HA (50, 100, 150 and 200 µg/mL) (R&D Systems Inc., MN, USA) for 6 hours in a humidified 5% CO_2 incubator at 37ºC. For the assessment of NFκβ intracellular signaling, PBMC, and THP1 cells were incubated in the presence of either vehicle (0.01% EtOH) or 10 µM of the proteasome inhibitor MG132 (Merck Millipore, MA, USA) for 30 min before the addition of either LMW HA (100 µg/ml and 100 pg/ml) or 100 ng/ml of the Toll-like receptor 2 (TLR2) agonist Pam2CSK4 (InvivoGen, CA, USA) for 2 and 6 hours. At the end of the incubation periods, cells were washed and collected for further gene expression analysis.

Human Adipose tissue explants and *ex vivo* incubations

Human visceral adipose tissue from obese patients was collected under sterile conditions and placed into a P100 culture plate with pre-warmed (37°C) $DPBS^{--}$ containing penicillin (100 U/ml)/streptomycin (100 µg/ml). Connective tissue and blood vessels were removed by dissection before cutting the tissue into small pieces (60–80 mg). Explants were washed with $DPBS^{--}$ at 37°C by centrifugation for 1 min at 400 *g* to remove blood cells and then maintained overnight in culture in Dulbecco's Modified Eagle Medium (DMEM) with L-glutamine (2 mM), antibiotics and 10% FBS. After a gentle rinse in $DPBS^{--}$ containing penicillin/streptomycin,

treatments were performed in DMEM with L-glutamine, antibiotics and 1% endotoxin-free BSA–fatty acid-free (FAF) (Sigma Aldrich, St Louis, MO, USA). Briefly, tissue explants were incubated with vehicle (0.01% EtOH) and the recombinant cytokines IL-1β (25 pg/ml), IL-6 (10 ng/ml) and IL-10 (20 ng/ml) (R&D Systems Inc., MN, USA) for 6 hours at 37ºC. At the end of the incubation period, tissue explants were frozen in liquid nitrogen, placed in polypropylene tubes, and stored at -80°C for further mRNA expression analysis of HAS1.

RNA isolation, reverse transcription and real-time PCR

Isolation of total RNA from adipose tissue, PBMC, PMN and THP-1 cells was performed using the TRIzol reagent (ThermoFisher Scientific, Waltham, MA, USA). RNA concentration was assessed in a NanoDrop-1000 spectrophotometer (NanoDrop Technologies, DE, USA), and its integrity tested with a RNA 6000 Nano Assay in a Bioanalyzer 2100 (Agilent Technologies, CA, USA). cDNA synthesis from 0.5 μg of total RNA was performed using the High-Capacity cDNA Archive Kit (Applied Biosystems, MA, USA). Real-time PCR analysis of HAS1 (Hs00987418_m1), HAS2 (Hs00193435_m1), HAS3 (Hs00193436_m1), HYAL1 (Hs00201046_m1), HYAL2 (Hs00234140_m1), HYAL3 (Hs00153133_m1), CD44 (Hs01075861_m1), LYVE-1 (Hs00272659_m1), TLR2 (Hs00152932_m1), TLR4 (Hs00152939_m1), IL-1β (Hs01555410_m1), IL-6 (Hs00985639_m1), IL-8 (Hs00174103_m1), CCL-2 (MCP-1) (Hs00234140_m1), cyclooxygenase (COX)-2 (Hs00153133_m1) and TNFα (Hs01113624_g1), was performed in a 7900HT Fast System (Applied Biosystems, MA, USA) using β-actin (Hs99999903_m1) as the endogenous control. PCR results were analyzed with Sequence Detector 2.1 software (Applied Biosystems, MA, USA). Relative quantification of gene expression was performed using the comparative Ct method. The amount of target gene, normalized to β-actin and relative to a calibrator, was determined by the arithmetic equation 2^{-}

$^{\Delta\Delta Ct}$, as described in the comparative Ct method (http://docs.appliedbiosystems.com/pebiodocs/04303859.pdf).

Western blot analysis

Total protein was extracted using a lysis buffer containing 50 mM HEPES, 20 mM β-glycerophosphate, 2 mM EDTA, 1% Igepal, 10% glycerol, 1 mM $MgCl_2$, 1 mM $CaCl_2$, and 150 mM NaCl for adipose tissue and a modified RIPA buffer containing 50 mM Tris-HCl, 150 mM NaCl, 1% Igepal, and 0.25% 1 mM EDTA for leukocytes and THP1 cells, both supplemented with protease and phosphatase inhibitors from Roche Diagnostics (Complete Mini and PhosSTOP cocktails, respectively). Homogenates were incubated on ice for 10 min and centrifuged at 14000 *g*, 15 min at 4 °C. Total protein (12 µg) from supernatants was placed in SDS-containing Laemmli sample buffer, heated for 5 min at 95°C, and separated by 10% SDS-PAGE for 120 min at 120 V. Transfer was performed by the iBlot Dry Blotting System (Invitrogen, MA, USA) onto PVDF membranes at 20 V over 7 min, and the efficiency of the transfer was visualized by Ponceau S staining. The membranes were then soaked for 1 h at room temperature in 0.1% T-TBS and 5% (w/v) non-fat dry milk. Blots were washed 3 times for 5 min each with 0.1% T-TBS and subsequently incubated overnight at 4°C with primary anti-human antibodies for phospho-IKK α/β (2697S, 1:1000; Cell Signaling Technology, MA, USA), IKK α/β (sc-7607,1:400; Santa Cruz Biotechnology, TX, USA) phospho-IKB-α (9246S, 1:200; Cell Signaling Technology, MA, USA) and IKB-α (sc-203, 1:200; Santa Cruz Biotechnology, TX, USA). Thereafter, the blots were washed 3 times for 5 min each with 0.1% T-TBS containing 5% (w/v) non-fat dry milk and incubated for 1 h at room temperature with either donkey anti-rabbit (Biolegend, CA, USA) or anti-mouse (Cell Signaling Technology, MA, USA) HRP-linked antibody (1:2000) in 0.1% T-TBS. Bands were visualized using the EZ-ECL chemiluminescence detection kit (Biological Industries, Israel). To assess housekeeping

protein expression, membranes were reblotted overnight at 4°C with primary rabbit anti-human β-actin HRP conjugate (Cell Signaling Technology, MA, USA) and detected and visualized as described above. Total IKK α/β, IKB-α and β-actin were used as internal controls when applicable to verify basal level expression and equal protein loading.

Luminex xMAP Technology

Cytokine levels were determined in plasma samples (25 μl) using a Milliplex MAP Human Cytokine/Chemokine Magnetic Bead Panel (Merck Millipore, MA, USA) on a Luminex 100 Bioanalyzer (Merck Millipore). The readouts were analyzed with the standard version of the Milliplex Analyst software (Merck Millipore). A five-parameter logistic regression model was used to generate standards curves (pg/mL) and to calculate the concentration of each sample.

Statistical analysis of the results was performed by analysis of variance for multiple comparisons (one-way or two-way ANOVA) or the unpaired Student's t test for single comparisons. An adjusted p-value ≤ 0.05 was considered statistically significant.

Supplementary TABLE 1. Spearman's rho correlation between plasma cytokine levels and body mass index (BMI) in n=48 subjects included in the study (*P*, p-value).

	r	*P* value
IL-1ra	0.69	0.0001
IL-6	0.57	0.0001
MCP-1	0.57	0.0001
IP-10	0.53	0.0001
IL-10	0.50	0.005
TNFα	0,44	0.005
IL-8	0.41	0.005
MIP-1β	0.33	0.05
IL-7	0.28	n.s.
VEGF	0.25	n.s

n=48 XY pairs

Supplementary Figure 1

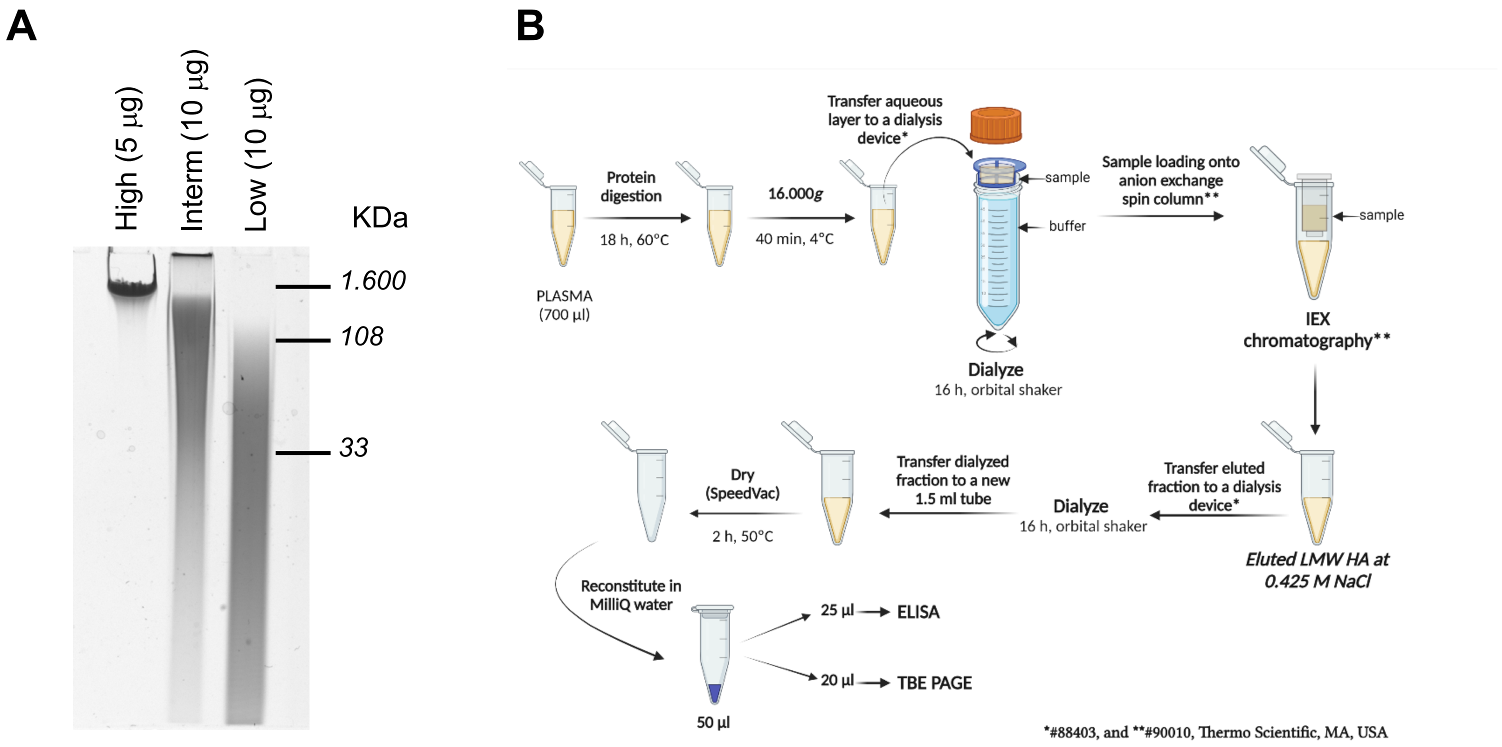

Supplementary Fig. 1. Plasma LMW HA isolation by IEX chromatography. (A) Representative polyacrylamide gel electrophoresis image of polydisperse hyaluronan commercial products of different molecular sizes (average kDa values shown on the right). High molecular weight (MW) HA (average mass of 1600 kDa), Intermediate MW HA (average mass of 108 kDa), and Low MW HA (average mass of 33 kDa). **(B)** Schematic diagram of plasma HA ion-exchange (IEX) chromatography protocol detailed at the *Material and Methods* included as *Supplementary information*. Created with Biorender.com.

Supplementary Figure 2

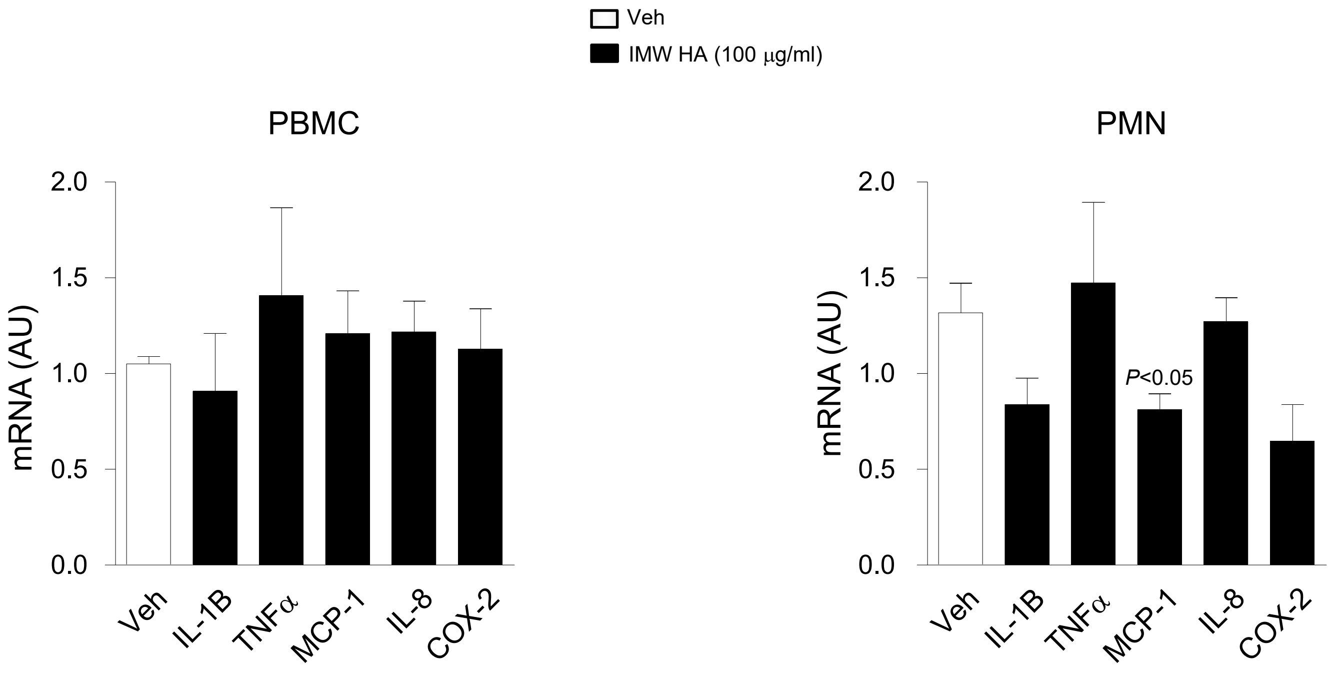

Supplementary Fig. 2. Intermediate MW HA effects on cytokine expression in leukocytes. Relative mRNA levels for IL-1β, TNFα, MCP-1, IL-8, and COX-2 in both human PBMC and PMN incubated for 6 h in the presence of vehicle (sterile water) or Intermediate MW HA (IMW HA, 75-350 kDa,100 μg/mL). Results are expressed as mean ± SEM of 3 independent experiments performed in duplicate.
